# Supplementary material for: Prevalence and Potential Determinants of COVID-19 Vaccine Hesitancy and Resistance in Qatar: Results from a Nationally Representative Survey of Qatari Nationals and Migrants between December 2020 and January 2021
Source: Vaccines (Basel). 2021 May 7;9(5):471. doi: 10.3390/vaccines9050471 (PMC8151168; doi:10.3390/vaccines9050471)
Supplement: Supplementary file 1 [file vaccines-09-00471-s001.zip › Supplementary Table S1.pdf]

Table S1: Models of the association between willingness to get COVID-19 vaccine and socio-demographics, work–and health-related characteristics

		Unadjusted model						Reduced model						Fully adjusted model					
		Hesitant			Resistant			Hesitant			Resistant			Hesitant			Resistant		
Variables	Ref	RRR	95%CI	p-value	RRR	95%CI	p-value	RRR	95%CI	p-value	RRR	95%CI	p-value	RRR	95%CI	p-value	RRR	95%CI	p-value
**Age group**	18-29																		
30-34		1.38	0.86-2.22	0.175	0.35	0.15-0.79	0.012	1.98	1.11-3.55	0.021	0.42	0.14-1.27	0.125	1.91	1.03- 3.57	0.041	0.40	0.13- 1.27	0.119
35-39		0.92	0.57-1.46	0.711	0.54	0.28-1.04	0.065	1.22	0.71-2.11	0.476	0.62	0.27-1.41	0.256	1.13	0.61- 2.08	0.696	0.53	0.21- 1.30	0.161
40+		0.77	0.52-1.15	0.199	0.59	0.33-1.03	0.063	0.86	0.53-1.40	0.557	0.58	0.29-1.17	0.129	0.82	0.45- 1.48	0.515	0.46	0.20- 1.04	0.063
**Gender**	Male											-							
Female		2.15	1.57-2.96	<0.0001	4.23	2.67-6.69	<0.0001	1.52	1.00-2.30	0.051	3.58	1.83-7.04	<0.0001	1.52	1.00- 2.30	0.049	3.73	1.89- 7.34	<0.0001
**Migrant Status/Type**	Qataris (non-Migrants)																		
White-collar Migrants		0.54	0.35-0.84	0.06	0.18	0.10-0.30	<0.0001	0.87	0.50-1.52	0.635	0.40	0.20-0.81	0.011	0.76	0.43- 1.34	0.348	0.37	0.18- 0.76	0.006
Blue-collar Migrants		0.28	0.16-0.47	<0.0001	0.11	0.05-0.25	<0.0001	0.55	0.28-1.07	0.077	0.37	0.14-0.97	0.044	0.49	0.25- 0.97	0.039	0.36	0.13- 1.00	0.050
**Language**	English																		
Arabic		3.21	2.40-4.31	<0.0001	5.48	3.24-9.26	<0.0001	3.10	2.11-5.56	<0.0001	3.76	1.73-8.14	0.001	3.06	2.08- 4.51	<0.0001	3.83	1.72- 8.56	0.001
**Education level**	Undergrad or less																		
Graduate/Prof		0.74	0.50-1.10	0.136	0 .31	0.14-0.69	0.005	0.71	0.45-1.10	0.122	0.36	0.13-1.01	0.051	0.73	0.47- 1.15	0.173	0.40	0.14- 1.15	0.090
**Employment status**	Unemployed																		
Employed		0.48	0.34-0.68	<0.0001	0.30	0.19-0.49	<0.0001	0.86	0.54-1.37	0.537	1.28	0.63-2.59	0.488	0.86	0.54- 1.37	0.523	1.25	0.62- 2.52	0.528
**Marital status**	Ever married																		
Never married		0.92	0.65-1.30	0.640	0.93	0.55-1.58	0.783	-	-	-	-	-	-	0.87	0.53- 1.44	0.599	0.83	0.41- 1.70	0.616
**Living arrangement**	Live with Others																		
Live Alone		0.50	0.34-0.73	<0.0001	0.48	0.24-0.95	0.035	0.68	0.43-1.08	0.104	1.18	0.53-2.64	0.682	0.69	0.43- 1.10	0.116	1.20	0.53- 2.72	0.670
**Depression or anxiety[1]**	No																		
Yes		1.74	0.98-3.09	0.060	2.71	1.30-5.63	0.008	1.04	0.53-2.05	0.909	1.00	0.41-2.43	0.994	1.01	0.51- 2.03	0.971	1.00	0.40- 2.51	0.996
**Chronic disease[2]**	No																		
Yes		0.90	0.65-1.24	0.520	1.45	0.91-2.31	0.119	-	-	-	-	-	-	0.80	0.53- 1.20	0.272	1.17	0.63- 2.13	0.621
**Quarantine status[3]**	No																		
Yes		0.96	0.68-1.35	0.813	0.86	0.51-1.44	0.566	-	-	-	-	-	-	0.75	0.48- 1.17	0.21	0.51	0.24- 1.09	0.081
**COVID-19 Infection status[4]**	No																		
Yes		0.99	0.59-1.66	0.975	1.24	0.60-2.54	0.563	-	-	-	-	-	-	-	-	-	-	-	-
**COVID-19 related death[5]**	No																		
Yes		1.18	0.78-1.78	0.430	0.72	0.36-1.44	0.355	-	-	-	-	-	-	1.21	0.75- 1.96	0.426	0.69	0.27- 1.75	0.435
**COVID-19 Infection Concerns[6]**	Not concerned at all																		
Somewhat/ Very concerned		0.90	0.66-1.24	0.530	0.49	0.31-0.79	0.003	0.95	0.65-1.38	0.770	0.41	0.22-0.76	0.005	0.95	0.65- 1.39	0.789	0.40	0.21- 0.75	0.004
**COVID-19 vaccine side-effects are of concern[7]**	Strongly disagree																		
Somewhat agree or disagree/ Neutral		4.43	2.50-7.85	<0.0001	0.19	0.09-0.42	<0.0001	4.34	2.19-8.59	<0.0001	0.28	0.10-0.80	0.017	4.50	2.26- 8.93	<0.0001	0.32	0.11- 0.91	0.033
Strongly agree		5.46	3.01-9.90	<0.0001	2.06	1.10-3.86	0.025	6.41	3.15-13.05	<0.0001	3.66	1.47-9.08	0.005	6.76	3.29- 13.86	<0.0001	4.05	1.61- 10.16	0.003

Note. Ref. is reference category; CI is 95% confidence intervals. Univariable model regresses one potential exploratory variable on the dependent variable - willingness to take the vaccine. Reduced model adjusts for significant variables from the univariable models (n=851). Fully adjusted model adjusts for all variables (n=849) with the exception of COVID-19 status as it was collinear with infection concerns (concerns about oneself/others contracting COVID-19). Willingness to get the vaccine was categorized into: accept (strongly agree), hesitant (somewhat agree, neutral, somewhat disagree), and resistant (strongly disagree).

[1] defined as a cut-off of 10 or more on Physician Health Questionnaire (PHQ-9) or the Generalized Anxiety Disorder Questionnaire (GAD-7); [2]Chronic disease defined by respondents' endorsement of one of the following conditions: diabetes, high blood pressure, high cholesterol, asthma, heart disease, cancer or cancerous tumors, and disability (physical, visual, hearing); [3]defined as any experience of quarantine since the pandemic stared [4]defined as positive if the respondent reported positive status confirmed by a test; [5]defined as death of someone close due to COVID-19;[6]measuring the extent of concern about themselves or their family members contracting COVID-19 (not-at-all concerned, not to concerned, somewhat concerned, very concerned);[7]defined as the degree of agreement/disagreement that "When a coronavirus vaccine becomes available, I will be concerned of its side effects"
