# Supplementary material for: Prevalence and Potential Determinants of COVID-19 Vaccine Hesitancy and Resistance in Qatar: Results from a Nationally Representative Survey of Qatari Nationals and Migrants between December 2020 and January 2021
Source: Vaccines (Basel). 2021 May 7;9(5):471. doi: 10.3390/vaccines9050471 (PMC8151168; doi:10.3390/vaccines9050471)
Supplement: Supplementary file 1 [file vaccines-09-00471-s001.zip › Supplementary Table S2.pdf]

Supplement Table S2. Willingness to get COVID-19 vaccine by socio-demographics, work-related, and health-related characteristics.

Variables		Willingness to get the vaccine[11] (%)			
		Accept	Hesitant	Resistant	p-value*
**Migrant Status/Type**	Qataris (non-migrants)	23.7	49.2	27.1	<0.0001
	White-collar Migrants	42.9	48.3	8.8	
	Blue-collar Migrants	58.5	33.8	7.7	
**Education level**	Undergraduate or less	41.4	45.5	13.1	0.010
	Graduate/Professional	52.3	42.6	5.1	
**Gender**	Male	50.0	41.9	50.0	<0.0001
	Female	28.6	51.6	28.6	
**Age group (years)**	18-29	39.2	43.5	17.3	0.003
	30-34	37.1	57.1	5.7	
	35-39	44.4	45.1	10.5	
	40+	47.3	40.4	12.2	
**Marital status**	Ever married	42.2	45.6	12.2	0.889
	Never married	44.1	44.0	11.9	
**Employment status**	Unemployed	28.1	52.7	19.3	<0.0001
	Employed	47.3	42.9	9.8	
**Ethnicity**	Arab	28.9	54.2	17.0	<0.0001
	Non-Arab	59.2	34.5	6.3	
**Living arrangement**	Live with others	39.8	47.5	12.8	0.001
	Live alone	57.2	34.1	8.8	
**Depression or anxiety[1]**	Yes	28.9	51.0	20.1	0.018
	No	44.0	44.7	11.3	
**Chronic disease[2]**	Yes	42.7	41.6	15.8	0.125
	No	42.8	46.3	10.9	
**COVID-19 status[3]**	Yes	41.8	43.8	14.4	0.819
	No	42.8	45.3	11.9	
**COVID-19 related death[4]**	Yes	41.1	50.2	8.7	0.337
	No	43.0	44.5	12.6	
**Quarantine status[5]**	Yes	44.0	45.0	11.0	0.848
	No	42.4	45.2	12.4	
**COVID-19 infection concerns[6]**	Not at all/not too concerned	38.9	44.5	16.6	<0.0001
	Somewhat concerned	35.2	54.8	10.0	
	Very concerned	53.4	37.8	8.8	
**Effective COVID-19 containment in Qatar[7]**	Strongly Agree	46.3	42.5	11.2	<0.0001
	Somewhat agree or disagree/Neutral	26.1	57.4	16.5	
**COVID-19 vaccine side-effects are of concern[8]**	Strongly agree	32.5	46.1	21.5	<0.0001
	Somewhat agree	44.4	53.6	2.0	
	Neutral	42.1	51.8	6.0	
	Somewhat disagree	48.9	46.1	5.1	
	Strongly disagree	63.2	16.5	20.3	
**COVID-19 vaccine should be mandatory[9]**	Strongly agree	85.2	13.5	1.3	<0.0001
	Somewhat agree	36.0	61.6	2.4	
	Neutral	22.9	71.3	5.8	
	Somewhat disagree	20.0	74.0	6.0	
	Strongly disagree	7.3	46.7	46.0	
**Endorsement source for vaccine[10]**	My doctor	50.4	43.1	6.5	<0.0001

Ministry of Health	55.8	41.7	2.6
WHO	50.1	44.5	5.4
Positive feedback from Friends/family	33.1	60.2	6.7
Scientific research	32.9	62.2	4.9
Other	38.7	43.7	17.6
I will not accept vaccine	1.0	25.8	73.3

Note. All reported percentages % are weighted. All rows add to 100%. *Probabilities (*p*-values) were derived from design-based *F* tests, a weighted Pearson chi square statistic corrected for complex sampling design. The null hypothesis tests the assumption that there is no association between each explanatory variable with COVID-19 vaccine willingness groups. A test of p-value less than 0.05 would mean that the null hypothesis of independence between the explanatory variable and COVID-19 vaccine willingness groups could be rejected. The corresponding inference in this case would support evidence of statistical association between the explanatory variable and COVID-19 vaccine willingness groups.[1] defined as a cut-off of 10 or more on Physician Health Questionnaire (PHQ-9) or the Generalized Anxiety Disorder Questionnaire (GAD-7); [2]Chronic disease defined by respondents' endorsement of one of the following conditions: diabetes, high blood pressure, high cholesterol, asthma, heart disease, cancer or cancerous tumors, and disability (physical, visual, hearing); [3]defined as positive if the respondent reported positive status confirmed by a test; [4] defined as death of someone close due to COVID-19;[5]defined as any experience of quarantine since the pandemic stared;[6] measuring the extent of concern about themselves or their family members contracting COVID-19 (not-at-all concerned, not to concerned, somewhat concerned, very concerned);[7] defined as the extent of agreement/disagreement that the measures taken in Qatar have been effective in controlling the spread of the virus;[8] defined as the degree of agreement/disagreement that "When a coronavirus vaccine becomes available, I will be concerned of its side effects";[9]defined as the degree of agreement/disagreement that "getting the coronavirus vaccine should be made mandatory";[10] based on the question "What would make you more confident in accepting the vaccine?" .
